# Relations between Recent Past Leisure Activities with Risks of Dementia and Cognitive Functions after Stroke

**DOI:** 10.1371/journal.pone.0159952

**Published:** 2016-07-25

**Authors:** Adrian Wong, Alexander Y. L. Lau, Eugene Lo, Michael Tang, Zhaolu Wang, Wenyan Liu, Nicole Tanner, Natalie Chau, Lorraine Law, Lin Shi, Winnie C. W. Chu, Jie Yang, Yun-yun Xiong, Bonnie Y. K. Lam, Lisa Au, Anne Y. Y. Chan, Yannie Soo, Thomas W. H. Leung, Lawrence K. S. Wong, Linda C. W. Lam, Vincent C. T. Mok

**Affiliations:** 1 Department of Medicine and Therapeutics, The Chinese University of Hong Kong, Hong Kong, China; 2 Therese Pei Fong Chow Research Centre for Prevention of Dementia, The Chinese University of Hong Kong, Hong Kong, China; 3 Department of Imaging and Interventional Radiology, The Chinese University of Hong Kong, Hong Kong, China; 4 Institute of Neuroscience and the Second Affiliated Hospital of Guangzhou Medical University and Key Laboratory of Neurogenetics and Channelopathies of Guangdong Province and Ministry of Education of China, Guangzhou, China; 5 Department of Neurology, Jinling Hospital, Nanjing University School of Medicine, Nanjing, China; 6 Department of Psychiatry, Lui Che Woo Institute of Innovative Medicine, The Chinese University of Hong Kong, Hong Kong, China; University of Glasgow, UNITED KINGDOM

## Abstract

**Background:**

Leisure activity participation has been shown to lower risks of cognitive decline in non-stroke populations. However, effects of leisure activities participation upon cognitive functions and risk of dementia after stroke are unclear. The purpose of this study is to examine the effects of recent past leisure activities participation upon cognitive functions and risk of incident dementia after stroke.

**Methods:**

Hospital-based, retrospective cohort study. 88 of 1,013 patients with stroke or TIA having no prestroke dementia were diagnosed to have incident poststroke dementia (PSD) 3–6 months after stroke. Regular participation (≥3 times per week) in intellectual, recreational, social and physical activities over the year before the index stroke was retrospectively recorded at 3–6 months after stroke.

**Results:**

Logistic regression analyses showed that regular participation in intellectual (RR 0.36, 95%CI 0.20–0.63) and stretching & toning physical exercise (0.37, 0.21–0.64) was significantly associated with a reduced risk of PSD after controlling for age, education, prestroke cognitive decline, stroke subtype, prior strokes and chronic brain changes including white matter changes, old infarcts and global atrophy. Results were similar in patients with past strokes in unadjusted models. Participation in increased number of activities in general (*r* = 0.41, *p*<0.01) and in intellectual (*r* = 0.40, *p*<0.01), recreational (*r* = 0.24, *p*<0.01), strenuous aerobic (*r* = 0.23, *p*<0.01) and mind-body (*r* = 0.10, *p*<0.01) activities was associated with higher poststroke Mini-mental State Examination scores in models adjusted for prestroke cognitive decline.

**Conclusions:**

Regular participation in intellectual activities and stretching & toning exercise was associated with a significantly reduced short-term risk of PSD in patients with and without recurrent strokes. Participation in greater number of recent past leisure activities was associated with better poststroke cognitive performance. Findings of this retrospective cohort study call for studies of activity intervention for prevention of cognitive decline in individuals at elevated risk of stroke.

## Introduction

Post stroke dementia (PSD) is a common complication of stroke that affects up to one-third of stroke patients.[1] Participation in physical and intellectual activities has been shown to lower the risks of cognitive decline and dementia in the general population.[2] In stroke patients, engagement in healthy lifestyles may reduce mortality after stroke.[3] Lifestyle modification is advocated as an important method for slowing cognitive decline in patients with PSD.[4] Although studies have shown that participation in *poststroke* leisure activities, in particular physical activities, might protect against poststroke cognitive decline,[5] little is known regarding the effects of recent *prestroke* leisure activities upon PSD and poststroke cognitive functions. The objective of this study is to investigate how recent leisure activities influence risk of PSD and poststroke cognitive functions.

## Materials and Methods

### Study design and participants

This is a hospital-based, retrospective cohort study. Participants were consecutive patients admitted to an acute stroke unit of a university affiliated hospital in Hong Kong for stroke and transient ischemic attack (TIA) between 1 January 2009 and 31 December 2010 and were subsequently recruited in the STRIDE (STroke Registry Investigating cognitive DEcline) study.[6] The STRIDE study is a hospital-based study recruiting 1,013 patients aimed to evaluate the mechanisms, risk factors and trajectory of cognitive functions after stroke and TIA. Detailed inclusion and exclusion criteria for the STRIDE study are published previously.[6] In brief, participants had stroke and TIA diagnosed according to standard criteria, had sufficient sensory, motor and language proficiency for completion of cognitive tests. Patients were excluded from the STRIDE study if they had significant aphasia, as defined by a score of 3 in the language score of the National Institute of Health Stroke Scale (NIHSS), clinically significant psychiatric symptoms such as active psychosis, and known history of dementia before the index stroke as ascertained by medical records or informants. Written informed consent was given by the patients to participate in the STRIDE study. Proxy informed consent was sought for patients deemed unable to give consent on their own, for example, those with severe cognitive impairment. The Joint Chinese University of Hong Kong—New Territories East Cluster Clinical Research Ethics Committee approved this study with retrospective data collection. Patient information was de-identified prior to analysis.

### Clinical assessment and ascertainment of incident PSD

Cognitive assessment was conducted by trained research psychologists between 3 and 6 months after admission of the index event. Demographic information and medical history, including vascular risk factors profile were collected from the electronic medical record of the hospital. Definitions of medical history and vascular risk factors are presented elsewhere.[6] Objective psychometric testing was performed using the validated Cantonese version of the Mini-Mental State Examination (MMSE).[7] The Clinical Dementia Rating (CDR)[8] were rated using all available information from clinical history, psychometric performance and functional level. Each patient was rated as having 0 –No cognitive symptoms; 0.5 –mild cognitive symptoms; and 1, 2 and 3 for mild, moderate and severe dementia, respectively. Special attention was made to differentiate whether functional impairment was due to cognitive or physical deficits.[9] Prestroke dementia was determined by a board-certified neurologist (V.M. or L.A.) using multiple sources of information including computerized medical records, collateral accounts and clinical information. Patients with prestroke dementia were excluded from the STRIDE study. Patients who received CDR ratings ≥1 at the study visit were further invited for clinical assessment by neurologists (V.M. or L.A.) for the diagnosis of dementia according to the Diagnostic and Statistical Manual of Mental Disorder, Fourth Edition, Text Revision (DSM-IV-TR).[10] As patients with prestroke dementia were excluded, incident PSD was determined as the diagnosis of dementia at 3 to 6 months poststroke.[6]

### Estimation of prestroke cognitive functions

Prestroke cognitive functions were estimated using the Chinese version of the Informant Questionnaire on Cognitive Decline in the Elderly (IQCODE) administered in the same clinical visit between 3 and 6 months after admission of the index event. For patients who attended the clinical visit alone, a phone interview was arranged with a close informant to assess the IQCODE. Informants were asked to rate the changes on 26 items examining memory or other cognitive functions over the 10 years preceding the index stroke using a 5-point Likert scale ranging from ‘much worse’ to ‘much improved’. Items scores are averaged with higher scores indicating worse cognitive decline. A score of ≥4 is suggestive of prestroke dementia as shown in a previous study conducted in Chinese stroke patients.[11]

### Evaluation of recent past leisure activities

Recent past leisure activity participation was defined as leisure activities participated over the past one year prior to index event recorded using a standardized leisure activity questionnaire developed from a focus group study involving local professionals and elderly people (Table 1).[12] Activities were categorized into *intellectual*, *social*, *recreational* and *physical* activities. Physical activities were further grouped as *strenuous aerobic exercise* (e.g. jogging), *mind-body exercise* (e.g. yoga, Tai-Chi) and *stretching & toning exercise* (e.g. walking). Using a self-reported questionnaire, the patient was asked to retrospectively report his or her activity participation during the year prior to the index admission. In 712 (70.3%) of the sample, the informant was also interviewed together with the patient and the activity report was based on a consensus made by both the patient and informant. For patients with dementia (i.e. CDR≥1) activity data reporting relied on the informant. An activity was considered engaged if it was performed ≥30 minutes continuously each time or cumulatively for ≥30 minutes within 24 hours. Regular activity participation was considered to be engagement of ≥3 times per week for most weeks over the year before the index event.

**Table 1:** Classification of Leisure Activities [12].

Intellectual Activities	Reading, using computer, playing board/card games, playing mahjong, gambling, investing, writing, drawing, painting, calligraphy, singing, playing musical instrument
Social Activities	Joining social centre, volunteering, going to museum/exhibitions/movie, meeting friends or relatives, attending religious activities
Recreational Activities	Listening to radio and music, watching TV, shopping, cooking, fishing, plants or pet keeping
Physical Activities	
Strenuous Aerobic Exercise	Running/jogging, swimming, cycling, hiking, dancing, playing ball games, dancing, martial arts
Mind-body Exercise	Playing QiGong and QiGong-like exercise, Luk Tung Kuen, Tai Chi, Yoga
Stretching & Toning Exercise	Slow walking, general stretching and toning exercise

### Neuroimaging acquisition and analysis

Non-contrast brain computed tomography (CT) was performed with a multidetector row clinical CT scanner for all patients upon arrival at the accident and emergency department of the hospital. For patients whose stroke subtype could not be classified based on CT, MRI was performed within one week of admission. MRI was performed using a 1.5-T scanner (Sonata, Siemens Medical, Erlangen, Germany) or a 3.0-T scanner (Achieva 3.0 T TX Series, Philips Medical System, Best, the Netherlands) using standard protocols with the following sequences: diffusion weighted imaging (DWI), axial gradient echo (GE), axial spin echo (SE) T1-weighted fast field echo (FFE), turbo spin echo (TSE) proton density (PD) and T2–weighted, axial FLAIR (Fluid Attenuated Inversion Recovery), and Time-of-Flight (TOF) MRA for 1.5-T MRI; and DWI, blood sensitive venous bold sequence, axial SE T1-weighted FFE, TSE T2–weighted, axial FLAIR and TOF MRA for 3-T MRI. Among the 1,013 included participants recruited in the STRIDE study, 510 (50.4%) had MRI. The following neuroimaging measures were recorded: 1) *White matter changes* (WMC) was rated on axial FLAIR MRI or CT with the Age-Related WMC Scale (ARWMC);[13] *presence of old infarcts* that were not relevant to the index event; and 3) *global brain atrophy*, defined by as 4^th^ quartile of the ventricular-brain ratio (VBR) measured on axial MRI or CT.[14] Presumably, these chronic brain changes had taken place before the index event and therefore these measures reflected the status of the brain in the recent period prestroke. Intraclass correlation coefficients for inter-modality agreement between 30 randomly selected pairs of CT and MRI and intra- and inter-rater agreement for WMC and VBR rating on CT and MRI were between 0.75 and 0.99.[6]

### Statistical analysis

Demographic, clinical, cognitive and activity data were compared between patients with and without incident PSD with independent sample *t* test or *χ*^2^ test as appropriate. Proportion of regular activity participation was compared between patients with and without PSD using the *χ*^2^ test. Binomial logistic regression models were constructed with regular participation vs. participation of <3 times/week in each activity category as the independent variable and incident PSD as the dependent variable. As decreased activity level may be a prodromal sign of dementia, models were adjusted for prestroke IQCODE along with age, years of education, stroke subtypes and TIA, prior stroke, ARMWC, presence of old infarcts and presence of global atrophy to take into account the influence of prestroke cognitive impairment and chronic brain changes upon the association between activity participation and PSD. The Hosmer-Lemeshow test was performed to evaluate goodness-of-fit of each multivariable model. Unadjusted univariable models with activity measures as the independent variable were repeated in the subset of patients with prior strokes (*n* = 184 including *n* = 23 PSD) to estimate the generalization of findings to those with prior strokes. Given the small sample size of the group with incident dementia among patients with prior strokes, only univariable models were performed in this subset of patients to avoid model over-fitting. To evaluate whether the number of activities influenced poststroke cognitive functions, partial correlation was calculated between MMSE and the total number of activities and for each activity category with estimate of prestroke cognitive decline (i.e., IQCODE) adjusted. To adjust for type I error due to multiple statistical computations, α was set at 0.01.

## Results

One thousand and thirteen patients were recruited into the STRIDE study. Twelve patients (1%) had missing data in recent activities participation and were excluded from the relevant analyses. Informants were available for 712 (70.3%) patients. Informants’ relationships to patients were spouse (32.9%), adult children or in-laws (34.4%), siblings (1.2%), other relatives (1.4%) and others (0.5%).

**Fig 1:**
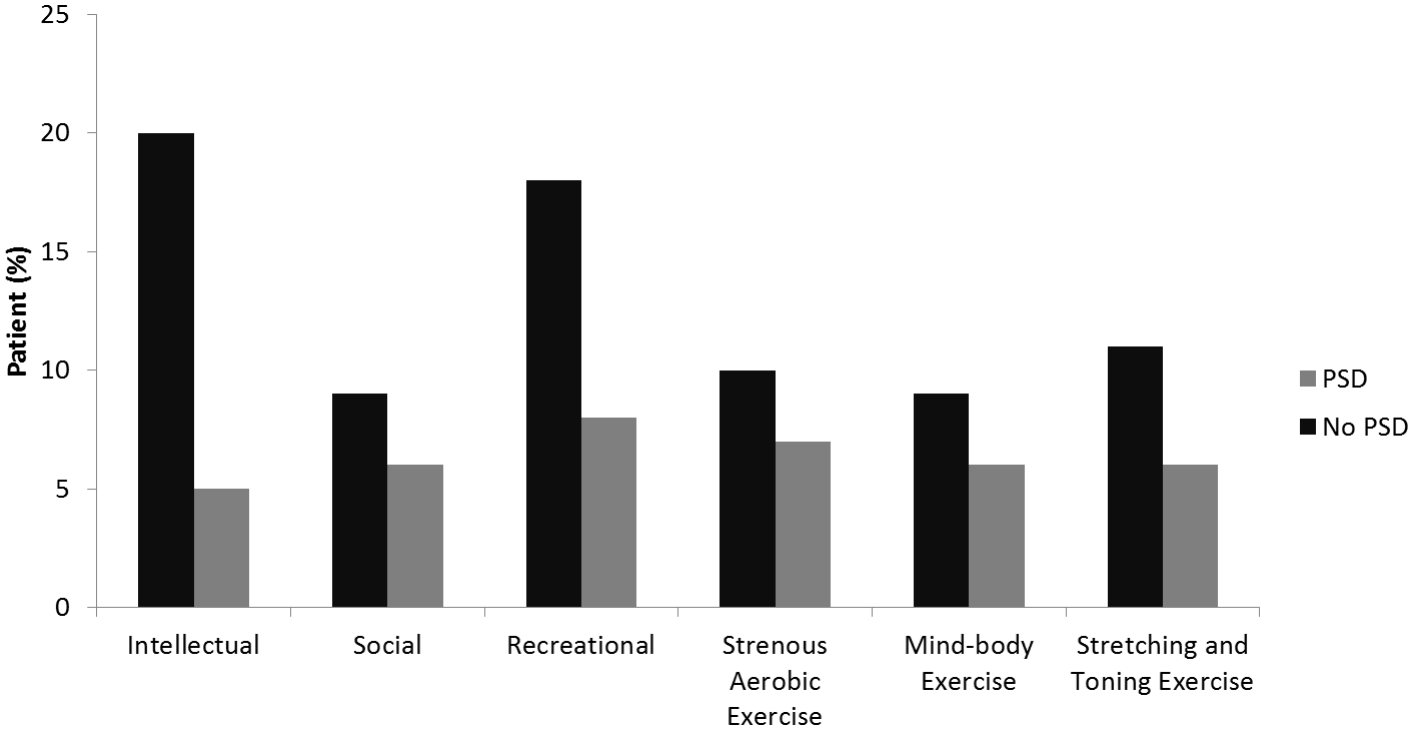
Comparison of regular participation in various activity categories in patients with and without PSD. *p<0.05 / **p<0.01 for group difference.

Eighty-eight patients (8.7%) were diagnosed to have incident PSD. Table 2 shows a comparison of the demographic, clinical, neuroimaging and activities data between patients with and without PSD. In summary, patients with PSD were older, predominately female, less educated, more likely to have hypertension, atrial fibrillation and congestive heart failure and had more severe strokes. They also had lower scores on the MMSE, higher prestroke IQCODE scores (indicating worse prestroke cognitive functions), more severe WMC and old infarcts and a higher frequency of global brain atrophy. Fig 1 shows the frequency of regular activity participation in patients with and without PSD. Compared to patients with PSD, those without PSD were more likely to have regularly participated in intellectual activities, stretching & toning exercise (*p*<0.01) and recreational activities (*p =* 0.035, trend difference).

**Table 2:** Comparisons between patients with and without incident PSD.

	Incident PSD		
	No	Yes	*p*
*n*	925 (91.3%)	88 (8.7%)	
*Demographic*			
Age in years	68.2 (11.4)	79.9 (9.0)	<0.001
Female	398 (43.0%)	51 (58.0%)	0.007
Education in years	5.8 (4.8)	3.2 (3.9)	<0.001
NIHSS at admission	4.3 (4.7)	9.3 (6.4)	<0.001
*Stroke subtypes and TIA*			
Large artery atherosclerosis	234 (25.3%)	24 (27.3%)	0.684
Small-artery occlusion	276 (29.8%)	15 (17.0%)	0.011
Cardioembolism	139 (15.0%)	21 (23.9%)	0.030
Intracerebral hemorrhage	58 (6.3%)	14 (15.9%)	0.001
Transient ischemic attack	135 (14.6%)	6 (6.8%)	0.044
Other stroke types	83 (9.0%)	8 (9.1%)	0.971
*Vascular risk factors*			
Hypertension	626 (67.7%)	72 (81.8%)	0.009
Diabetes mellitus	316 (34.2%)	41 (46.6%)	0.024
Hyperlipidemia	556 (60.1%)	42 (47.7%)	0.015
Smoking	333 (36.0%)	25 (28.4%)	0.167
Alcohol drinking	124 (13.4%)	9 (10.2%)	0.388
Prior stroke or TIA	180 (19.5%)	24 (27.3%)	0.037
Atrial fibrillation	143 (15.5%)	24 (27.3%)	0.005
Ischemic heart disease	80 (8.6%)	11 (12.5%)	0.243
Congestive heart failure	26 (2.8%)	9 (10.2%)	<0.001
*Cognitive functions*			
Pre-stroke IQCODE#	3.08 (0.28)	3.37 (0.81)	<0.001
MMSE	25.1 (4.7)	12.9 (5.5)	<0.001
*Chronic brain changes*			
ARWMC scale total score	3.2 (3.9)	6.1 (4.3)	<0.001
Total number of old infarcts	1.73 (2.4)	3.06 (3.8)	<0.001
Presence of global atrophy*	204 (22.1%)	49 (55.7%)	<0.001

Abbreviations: NIHSS-National Institute of Health Stroke Scale; TIA-Transient Ischemic Attack; MMSE-Mini-Mental State Examination; IQCODE-Informant Questionnaire on Cognitive Decline in the Elderly; ARWMC-age related white matter changes;

* presence of global atrophy defined as ≥ 4^th^ quartile of ventricle-brain ratio

^#^Data shown in median (interquartile range).

**Fig 2:**
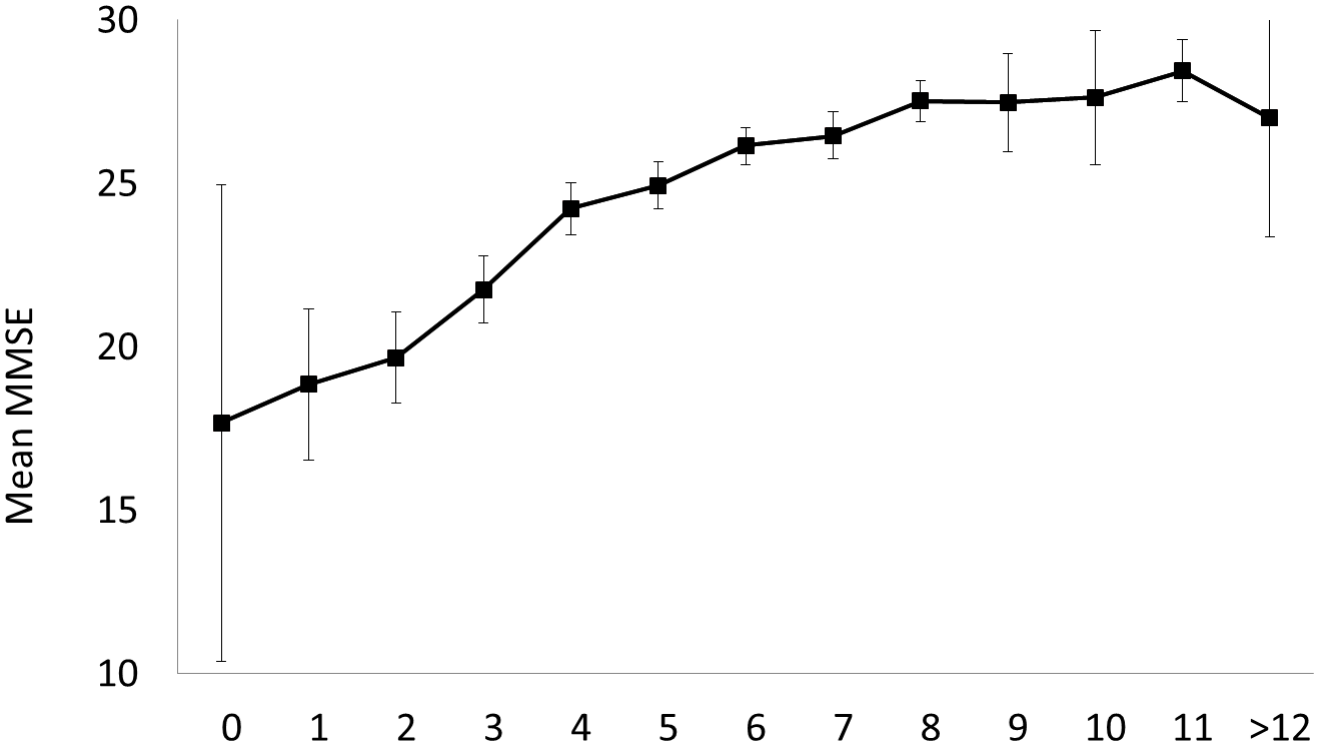
Relationship between total numbers of leisure activities regularly participated and poststroke MMSE controlling for prestroke IQCODE. (*r* = 0.41, *p*<0.01). Error bars are 95% confidence interval.

Table 3 shows the results of the logistic regression models examining the effects of recent past leisure activities upon risk of PSD. In the whole sample, multivariable models adjusted for demographic, clinical and neuroimaging variables revealed that regular participation in intellectual and stretching & toning exercise was significantly associated with reduced risks of PSD. All multivariable models fit the observed data (*p* for Goodness-of-fit test >0.40 for all models). Unadjusted analysis in patients with prior strokes showed similar findings. In contrast, social, recreational and other types of physical activities did not significantly attenuate the risk of incident PSD. After adjusting for the effects of prestroke cognition, participation in increased number of activities in general (*r* = 0.41, *p*<0.01; Fig 2) and in intellectual (*r* = 0.40, *p*<0.01), recreational (*r* = 0.24, *p*<0.01), strenuous aerobic (*r* = 0.23, *p*<0.01) and mind-body (*r* = 0.10, *p*<0.01) activities was associated with higher MMSE performance poststroke.

**Table 3:** Binomial logistic regression models examining effects on regular activity participation upon risk of incident PSD.

	All patients				Patients with prior strokes only	
	Total *n* = 1,013				Total *n* = 184	
	Incident dementia = 88				Incident dementia = 23	
	Unadjusted Model		Adjusted Model		Unadjusted Model	
Activity Category	RR	95% CI	RR	95% CI	RR	95% CI
Intellectual	0.24	0.15 to 0.37**	0.36	0.20 to 0.63**	0.13	0.05 to 0.33**
Social	0.70	0.25 to 1.97	0.58	0.19 to 1.73	0	0 to 0
Recreational	0.41	0.18 to 0.90	0.42	0.15 to 1.16	0.27	0.05 to 1.55
Strenuous aerobic exercise	0.69	0.35 to 1.37	1.32	0.62 to 2.85	1.05	0.33 to 3.32
Mind-body exercise	0.64	0.27 to 1.51	0.60	0.24 to 1.52	0.25	0.03 to 1.92
Stretching & toning exercise	0.46	0.29 to 0.73**	0.37	0.21 to 0.64**	0.23	0.08 to 0.64**

Relative Risk (RR) denotes risk of regular participation (≥3 times/week) vs. <3times/week in incident PSD.

Model adjusted for age, years of education, prestroke IQCODE, stroke subtypes, prior strokes, ARWMC, presence of old infarcts, presence of global atrophy entered as covariates.

** *p<*0.01.

## Discussion

In this study we showed that participation in ≥3 times per week in recent past intellectual activities and stretching & toning exercise was associated with a reduced risk of incident PSD in the short-term (i.e. between 3 to 6 months) after stroke or TIA. Moreover, similar effects were observed in patients with prior strokes. A dose-benefit relationship was found between the numbers of leisure activities participated in general and in intellectual, social, recreational activities, strenuous aerobic and mind-body exercises with poststroke cognitive performance.

Engagement in physical and intellectual activities has demonstrated cognitive benefits in large-scale observational studies in the general elderly population and among patients with cognitive and vascular disorders.[15, 16] Benefits of leisure activity participation upon cognition are believed to be mediated by increasing brain and cognitive reserve.[17] Physical activities are recommended by the American Stroke Association for primary prevention of stroke,[18] with moderate to high intensity physical activity believed to be most beneficial.[19] Engagement in moderate to high intensity physical activity was associated with better daily functioning and lower level of physical handicap three months poststroke.[20] However, there is a paucity of data regarding the relationship between engagement in recent past leisure activities and risks of PSD, and the effect of exercise intensity upon cognition is less clear. In this study, engagement in strenuous aerobic exercise was associated with higher poststroke MMSE scores but not a lower risk of PSD. Instead, stretching & toning exercise such as walking was associated with a risk reduction of approximately 63%. Note that in our sample with high cerebrovascular risk burden, the level of participation in physical exercise appeared low when compared to those reported in community studies.[21] However, these findings suggest that even low level of stretching & toning exercise in a high-risk group was associated with a lower risk of developing dementia after stroke. Stretching & toning exercises are popular among older adults and highly feasible even for those with physical and cognitive impairment. It is possible that stretching & toning exercise contributes to neuroplasticity by mechanisms that strenuous aerobic exercises do not share but this postulation warrants further investigation. The lack of relationship between aerobic exercise and PSD may be explained by the possibility that people who regularly engaged in strenuous aerobic exercise might have prevented having the stroke in the first place. In addition to effects upon risk of PSD, we showed that recent participation in greater number of intellectual, recreational, strenuous aerobic exercise and mind-body exercise were modestly but significantly associated with better poststroke MMSE performance. Such results suggest that these activities might still benefit cognitive functions poststroke. Overall, our findings advocate a multimodality activity intervention with integration of physical, intellectual, social and recreational activities to prevent cognitive decline in stroke patients.[22]

### Study Limitations

The strength of our study included a well-defined cohort of a large sample of patients with stroke and TIA. Missing data were also minimal. However, there are a number of limitations in this study. First and foremost, as a retrospective cohort study, evaluation of activity participation was based on retrospective self-report that might be subject to recall bias, especially among patients with cognitive impairment. Although we attempted to circumvent this limitation by obtaining consensus between patient and informant in the majority (70.3%) of the sample, the possibility of reporting bias by informant for patients with dementia could not be eliminated. Likewise, inaccuracy in recall and medical records could have impacted the correct assessment of prestroke cognitive status. Also, as some studies suggested that midlife activities and more years of participation might confer larger cognitive benefits than late life activities,[23, 24] we only assessed activities participated during the year prior to index admission. Similarly, effects of non-leisure activities such as level of work complexity were not considered in this study. Moreover, our findings only apply to patients without prestroke dementia and thus it is not clear whether and to what extent activity participation may influence cognitive decline in patients who have already had dementia before the stroke. At the same time, we might have inadvertently included patients with prestroke dementia as the assessment relied on proxy recall of patient history and medical records. Furthermore, despite the adjustment for stroke subtypes in the analysis, our findings may mostly apply to patients with mild strokes or TIA and thus further investigations should be conducted in patients with more severe strokes such as those with intracranial hemorrhage or cardioembolic strokes. Finally, although we tried to exclude patients with prestroke dementia, those with PSD had more prestroke cognitive decline and chronic brain changes compared to those without PSD. With a retrospective study design, it can be argued that people heralding cognitive decline and brain pathology prestroke may engage in fewer activities and thus it is difficult to attribute any risk reduction to activity participation (note however a study showed that cognitive activity participation was not correlated with Alzheimer’s disease or vascular neuropathology[25]). In view of this, we attempted to minimize the effects of prestroke cognitive status and chronic brain changes upon the associations between activity participation and poststroke cognition (PSD and MMSE) by adjusting for prestroke IQCODE and relevant MRI measures in the statistical models. Longitudinal studies on stroke-free persons at high risk of stroke are needed to substantiate our study findings.

## Conclusions

In conclusion, we showed that engagement in three or more times per week in intellectual activities and stretching & toning exercise one year prestroke was associated with a significant reduction in the risk of incident PSD 3 to 6 months after stroke or TIA. Regular participation in greater number of leisure activities was associated with better poststroke cognitive performance. However, because of the retrospective nature of this study, our findings may only serve the purpose of hypothesis generation for future studies in activity intervention for prevention of cognitive impairment in populations at high risk of stroke.

## Supporting Information

S1 Dataset The minimal dataset *PONE-D-16-00200*.*sav* contains all the data pertinent to the analysis and results reported in this manuscript. (SAV)
